# Quinolizidine Alkaloid Biosynthesis in Lupins and Prospects for Grain Quality Improvement

**DOI:** 10.3389/fpls.2017.00087

**Published:** 2017-01-31

**Authors:** Karen M. Frick, Lars G. Kamphuis, Kadambot H. M. Siddique, Karam B. Singh, Rhonda C. Foley

**Affiliations:** ^1^Commonwealth Scientific and Industrial Research Organisation Agriculture and Food, Commonwealth Scientific and Industrial Research OrganisationFloreat, WA, Australia; ^2^School of Plant Biology, The University of Western AustraliaCrawley, WA, Australia; ^3^The UWA Institute of Agriculture, The University of Western AustraliaPerth, WA, Australia

**Keywords:** grain improvement, grain legume, lupin, *Lupinus angustifolius*, plant secondary metabolism, pulse, quinolizidine alkaloids

## Abstract

Quinolizidine alkaloids (QAs) are toxic secondary metabolites found within the genus *Lupinus*, some species of which are commercially important grain legume crops including *Lupinus angustifolius* (narrow-leafed lupin, NLL), *L. luteus* (yellow lupin), *L. albus* (white lupin), and *L. mutabilis* (pearl lupin), with NLL grain being the most largely produced of the four species in Australia and worldwide. While QAs offer the plants protection against insect pests, the accumulation of QAs in lupin grain complicates its use for food purposes as QA levels must remain below the industry threshold (0.02%), which is often exceeded. It is not well understood what factors cause grain QA levels to exceed this threshold. Much of the early work on QA biosynthesis began in the 1970–1980s, with many QA chemical structures well-characterized and lupin cell cultures and enzyme assays employed to identify some biosynthetic enzymes and pathway intermediates. More recently, two genes associated with these enzymes have been characterized, however, the QA biosynthetic pathway remains only partially elucidated. Here, we review the research accomplished thus far concerning QAs in lupin and consider some possibilities for further elucidation and manipulation of the QA pathway in lupin crops, drawing on examples from model alkaloid species. One breeding strategy for lupin is to produce plants with high QAs in vegetative tissues while low in the grain in order to confer insect resistance to plants while keeping grain QA levels within industry regulations. With the knowledge achieved on alkaloid biosynthesis in other plant species in recent years, and the recent development of genomic and transcriptomic resources for NLL, there is considerable scope to facilitate advances in our knowledge of QAs, leading to the production of improved lupin crops.

## Introduction

Quinolizidine alkaloids (QAs) are secondary metabolites that occur mostly within the family Leguminosae and they can occur in the genus *Lupinus*, as well as in *Baptisia, Thermopsis, Genista, Cytisus, Echinosophora*, and *Sophora* (Ohmiya et al., 1995). Whilst QAs offer the plants protection against insect pests (Wink, 1992; Berlandier, 1996; Wang et al., 2000; Philippi et al., 2015), they cause a concern for the human consumption of lupin grain and lupin-based foods as high levels confer a bitter taste and may result in acute anticholinergic toxicity, characterized by symptoms such as blurry vision, headache, weakness, and nausea (Daverio et al., 2013). The lethal dose of QAs in children is estimated to be 11–25 mg total alkaloids kg^-1^ body weight, while no fatal poisonings have been reported in adults (Allen, 1998; Petterson et al., 1998).

*Lupinus* is a diverse genus, though only four species have been domesticated and are agriculturally significant: *L. angustifolius* (NLL), *L. albus* (white lupin), *L. luteus* (yellow lupin), and *L. mutabilis* (pearl lupin; Petterson et al., 1998). These species have been domesticated relatively recently (Cowling et al., 1998) and as a consequence of this, undesirable traits such as the accumulation of QAs remain. While the grain has been used traditionally as an animal feed, it has gained recognition as a health food; it is high in protein and fiber and possesses certain beneficial nutraceutical properties (Petterson et al., 1997; Duranti et al., 2008; Sweetingham et al., 2008). QAs complicate the use of the grain for higher-value food purposes as they must remain below the industry threshold of 0.02% in Australia and some European countries (Cowling et al., 1998; Boschin et al., 2008; Jansen et al., 2009). QA levels can vary considerably from year to year under field conditions, often exceeding this threshold (Cowling and Tarr, 2004). As such, an understanding of the QA biosynthetic pathway is essential in assisting lupin breeders and farmers to produce high-value crops consistently.

Quinolizidine alkaloid biosynthesis has been studied far less extensively than some economically important alkaloids in other plant species, for example nicotine in *Nicotiana*, MIAs in *Catharanthus roseus*, i.e., vinblastine and vincristine, and BIAs in *Coptis japonica* and *Papaver somniferum*, i.e., berberine and morphine, respectively, which represent model species for understanding alkaloid biosynthesis. The past couple of decades have resulted in the identification of many genes involved in alkaloid biosynthesis in these species including biosynthetic genes, transcription factors and transporters, and the identification of enzymes and pathway intermediates through the development of genomic, transcriptomic, proteomic, and metabolomic data sets (Dewey and Xie, 2013; Hagel and Facchini, 2013; Beaudoin and Facchini, 2014; Pan et al., 2016). In the case of QAs, while the chemistry has been well characterized with more than 170 structures identified (Wink, 1993), the QA biosynthetic pathway has only been partially elucidated and information on enzymes and genes involved in QA biosynthesis is limited. Here, we discuss what is currently known about QA biosynthesis in lupin, draw on examples from model alkaloid species, and suggest future directions and ways to improve QA biosynthesis in lupin to produce higher-value lupin crops.

## Quinolizidine Alkaloids and Biosynthesis

Quinolizidine alkaloids are so-called because of their quinolizidine ring structure and can be divided into major structural classes: lupanine, angustifoline, lupinine, sparteine, multiflorine, aphylline, anagyrine and cytisine, though the latter two are usually absent in lupins and more commonly found in *Thermopsis, Sophora, Echinosophora*, and *Genista* (Wink, 1987a; Ohmiya et al., 1995; Boschin and Resta, 2013). Each lupin species has a characteristic alkaloid profile (**Table 1**). Usually, only the presence of major QAs are reported—defined as individual QAs with levels ≥1% of total QAs—although many other QAs have been detected at trace levels in each of the lupin species (Wink et al., 1995). Of the major QAs in lupin grain, three of the four domesticated lupins share lupanine and 13α-hydroxylupanine. Each cultivated lupin species also has unique major QAs such as isolupanine and angustifoline for NLL, albine and multiflorine for *L. albus*, and lupinine for *L. luteus* (**Table 1**). The indole alkaloid gramine is also a major component in bitter *L. luteus* grain and the piperidine alkaloid ammodendrine is found in major quantities in *L. mutabilis* and minor quantities in *L. luteus* and *L. albus* grain (Wink et al., 1995; de Cortes Sánchez et al., 2005; Adhikari et al., 2012). QAs vary in their toxicity and their deterrence against insect pests. Sparteine and lupanine appear to be the two most toxic QAs to humans and laboratory animals (Allen, 1998; Petterson et al., 1998), with lupanine having the greatest impact on aphid survival, followed by indole alkaloid gramine, sparteine, lupinine, and 13α-hydroxylupanine and angustifoline having the least impact (Ridsdill-Smith et al., 2004).

**Table 1:** Major QAs (≥1%) identified in seed of lupin species.

Lupin species	Major grain QAs (% of total alkaloids)	Reference
*L. angustifolius*	Lupanine (70%), 13α-hydroxylupanine (12%), angustifoline (10%)	Wink et al., 1995
	Lupanine (50.6%), 13α-hydroxylupanine (32.6%), angustifoline (10.4%), isolupanine (6.4%; average values)	Priddis, 1983
*L. albus*	Lupanine (70%), albine (15%), 13α-hydroxylupanine (8%), multiflorine (3%)	Wink et al., 1995
*L. luteus*	Lupinine (60%), sparteine (30%), unknown minor alkaloids (1%)	Wink et al., 1995
	Gramine^∗^ (10–89%), lupinine (3–60%), sparteine (1–20%), isosparteine (1–5%)	Adhikari et al., 2012
*L. mutabilis*	Lupanine (46%), sparteine (16%), 3β-hydroxylupanine (12%), 13α-hydroxylupanine (7%), ammodendrine^∗^ (2%), 13-angeloyloxylupanine (2%), tetrahydrorhombifoline (2%), 11-12-dehydrosparteine (1%) angustifoline (1%), 13-tigoyloxylupanine (1%)	Wink et al., 1995

*^∗^Gramine and ammodendrine are indole and piperidine alkaloids, respectively.*

**FIGURE 1:**
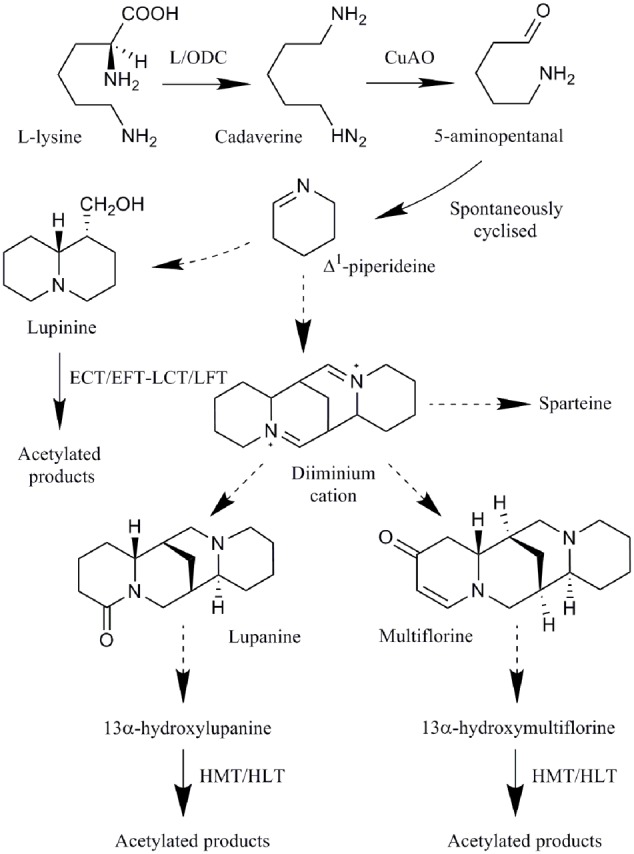
**Quinolizidine alkaloid biosynthetic pathway: all QAs are derived from lysine.** Enzymes involved in the pathway are L/ODC, copper amine oxidase (CuAO), and two acyltransferases (ECT/EFT-LCT/LFT and HMT/HLT). Dotted lines represent uncharacterized enzyme reactions (adapted from Bunsupa et al., 2012b).

The biosynthesis of all QAs begins with the decarboxylation of L-lysine to form the intermediate cadaverine by a L/ODC such as the *Lupinus angustifolius* L/ODC *(La-L/ODC*; Leistner and Spenser, 1973; Bunsupa et al., 2012a) (**Figure 1**). Cadaverine then undergoes oxidative deamination, by a copper amine oxidase (CuAO), to yield 5-aminopentanal which is then spontaneously cyclized to Δ^1^-piperideine Schiff base (Leistner and Spenser, 1973; Golebiewski and Spenser, 1988; Bunsupa et al., 2012b). It has been suggested that in addition to these reactions, a series of reactions including Schiff base formations, aldol-type reactions, hydrolysis, oxidative deamination and coupling gives rise to the major structural QAs (e.g., lupanine and others; Dewick, 2002), with the diiminium cation proposed as an intermediate in the biosynthesis of tetracyclic alkaloids (e.g., lupanine, multiflorine, and sparteine; Fraser and Robins, 1984). These QAs can then be further modified by dehydrogenation, oxygenation, hydroxylation, glycosylation, or esterification to form a wide variety of structurally related QAs (Wink and Hartmann, 1982a; Saito et al., 1993; Ohmiya et al., 1995). The acyltransferase HMT/HLT forms acetylated products of 13α-hydroxylupanine and 13α-hydroxymultiflorine and a *L. albus HMT/HLT* (*LaHMT/HLT*) gene encoding this enzyme has been characterized (Saito et al., 1992; Suzuki et al., 1994; Okada et al., 2005). The acyltransferase ECT/EFT-LCT/LFT forms acetylated products of lupinine and epilupinine (Saito et al., 1992, 1993; Suzuki et al., 1994; Bunsupa et al., 2012b). *L. angustifolius acyltransferase* (*LaAT*) is suggested to be involved in the formation of QA esters, though its enzymatic function has not been confirmed (Bunsupa et al., 2011).

While only two genes have been identified in QA biosynthesis, the discovery of biosynthetic genes involved in the formation of other alkaloids may assist in identifying homologous genes in the QA pathway, for example, *La-L/ODC* was identified as a homolog of *ODC*, involved in the biosynthesis of a precursor for nicotine biosynthesis (Bunsupa et al., 2012a). Enzymes common in nicotine, MIA, BIA, as well as Amaryllidaceae alkaloid biosynthetic pathways include: methyltransferases, decarboxylases, oxidases, acyltransferases, cytochromes P450 (cP450s), oxidoreductases, demethylases, reductases, hydroxylases and coupling enzymes, and genes encoding many of these enzymes have been identified in *Nicotiana, C. roseus, C. japonica*, and *P. somniferum* (Bird et al., 2003; Dewey and Xie, 2013; Hagel and Facchini, 2013; Kilgore and Kutchan, 2016; Pan et al., 2016; Thamm et al., 2016). Many of these common types of enzymes are either known (i.e., decarboxylase, oxidase, and acyltransferases) or suggested (listed above) to play a role in QA biosynthesis. Transcriptome analysis has also identified several genes co-expressed with a putative *Sophora flavescens L/ODC*, encoding a major latex-like protein (MLP-like), a cP450, a ripening related protein and an uncharacterized protein (Han et al., 2015), which may also have roles in QA biosynthesis. MLP-like proteins may be involved in BIA biosynthesis, though their biological function is unkown, and the berberine bridge and berberine bridge-like enzymes catalyze oxidative reactions for the biosynthesis of BIAs and *Nicotiana* alkaloids (Facchini et al., 1996b; Samanani et al., 2004; Kajikawa et al., 2011), possibly having similar roles in QA biosynthesis. Cytochromes P450 have a role in hydroxylation reactions, as well as other reactions, in MIA and BIA biosynthesis (Pauli and Kutchan, 1998; Thamm et al., 2016) and may be involved in QA hydroxylation reactions in the synthesis of derivatives of major structural QAs (**Figure 1**).

## Localization of QA Biosynthesis and Transport of QAs

There is strong evidence for the synthesis of QAs in aerial tissues of lupin as opposed to roots: lupin L/ODC is localized to chloroplasts (Wink and Hartmann, 1982b; Bunsupa et al., 2012a), *La-L/ODC* transcript level is highest in young leaves of bitter NLL, while barely detectable in mature leaves, cotyledons, hypocotyls and roots (Bunsupa et al., 2012a), cadaverine is incorporated into lupanine in aerial tissue but not in roots (Wink, 1987b) and grafting experiments in lupin, whereby high-QA lupin scions are grafted onto low-QA lupin roots and vice versa, show that shoots are more important than roots in determining overall plant QA content (Waller and Nowacki, 1978; Lee et al., 2007). The last step of lysine biosynthesis also takes place in the chloroplast (Mazelis et al., 1976; Wink and Hartmann, 1982b). Interestingly, *Lycopodium clavatum* L/ODC, with a role in biosynthesis of *Lycopodium* alkaloids which are also derived from lysine, is localized in the cytosol (Bunsupa et al., 2016) and perhaps the chloroplastic location of La-L/ODC increases its accessibility to lysine, rather than ornithine, as a substrate for the production of QAs.

The expression of *LaHMT/HLT* and HMT/HLT activity was associated with roots and hypocotyls of *Lupinus* plants (Saito et al., 1992; Okada et al., 2005), and the activity of both HMT/HLT and ECT/EFT-LCT/LFT was not associated with chloroplasts (Suzuki et al., 1996). This suggests that while the most important steps in the QA biosynthetic pathway take place in aerial tissues, it is possible that the entire pathway is not limited to such tissues. Once synthesized, QAs are then translocated to the reproductive organs via the phloem (Wink and Witte, 1984; Lee et al., 2007). The loading of QAs onto the phloem may be selective as lupin leaves have more diverse QA profiles than both grain and phloem exudates (Wink et al., 1995; Lee et al., 2007). No studies have yet investigated QA biosynthesis within seeds themselves. It has been estimated, based on measures of translocation of QAs and total QAs in reproductive tissues, that of the QAs that accumulate in seeds, half are synthesized within the seed and half are translocated (Lee et al., 2007).

The identification of sites of QA biosynthesis and transport processes is important for targeting the accumulation of QAs in grain. If QA biosynthesis within seeds themselves is not appreciable, this offers the means to target QA transport processes in order to reduce grain QA levels without compromising QA biosynthetic processes, which negatively affects plant fitness. In lupin, sweet (low QA) cultivars have considerably lower resistance to disease and predation compared to bitter (high QA) wild germplasm, increasing susceptibility to insect attack and transmission of aphid-borne viruses (Berlandier, 1996; Wang et al., 2000; Adhikari et al., 2012). In particular, sweet *L. luteus* varieties, which are valued because of their very high protein content, are susceptible to aphid attack and as such, are unsuccessful in Australia and resistance may be difficult to achieve with grain QA levels below 0.02% (Berlandier and Sweetingham, 2003; Adhikari, 2007). One concept for lupin breeding is to develop a ‘bitter/sweet’ phenotype*—*a plant that has sufficiently high QA levels in vegetative tissues to deter insect attack, but contains low QA levels in grain (Wink, 1990, 1994). For this, transporters involved in the translocation of QAs from source tissues to seeds must be identified.

Though several genes that are involved in the transport of nicotine, BIAs and a MIA precursor have been identified, no targets are yet identified which affect the alkaloid levels in source and sink tissues separately. In *Nicotiana*, nicotine is synthesized in roots and is usually transported to leaves *via* the xylem (Dawson, 1942; Baldwin, 1989). Transporters involved in the sequestration of nicotine into vacuoles belong to the multidrug and toxic compound extrusion (MATE) family (NtMATE1, NtMATE2, NtJAT1, NtJAT2; Morita et al., 2009; Shoji et al., 2009; Shitan et al., 2014) and a plasma membrane located, nicotine importer belongs to the purine uptake permease-like (PUP-like) family (NtNUP1; Hildreth et al., 2011). These nicotine transporters are mainly expressed in roots (*NtMATE1, NtMATE2, NtNUP1*), with *NtJAT1* expressed in all tissues and *NtJAT2* expressed almost exclusively in leaves and all are induced by methyl jasmonate (Morita et al., 2009; Shoji et al., 2009; Hildreth et al., 2011; Kato et al., 2014; Shitan et al., 2014). NtJAT1 may also function as a plasma membrane localized nicotine eﬄux transporter when produced in root tissue, suggesting that this transporter plays more than one key role in nicotine transport (Morita et al., 2009). Most of these MATE transporters also efficiently transport tropane alkaloids, with NtJAT1 and NtJAT2 additionally found to transport berberine, and NtNUP1 also transports vitamin B6 (Morita et al., 2009; Shoji et al., 2009; Hildreth et al., 2011; Shitan et al., 2014; Kato et al., 2015). Down-regulation of *NtMATE1/MATE2* transcript levels in *Nicotiana* plants using RNA-interference (RNAi) did not affect alkaloid levels in the leaves or the roots, however, did increase sensitivity of the plant to exogenously applied nicotine (Shoji et al., 2009). Down-regulation of *NtNUP1* reduced nicotine accumulation throughout the entire plant, however, root to shoot translocation was unaffected (Hildreth et al., 2011; Kato et al., 2014). Interestingly, *NtNUP1* positively regulates the expression of a key transcription factor in the nicotine biosynthesis pathway, possibly explaining the reduced nicotine content in RNAi lines (Kato et al., 2014). It would be interesting to assess the effect of down-regulating *NtJAT1* and *NtJAT2*, as these function as nicotine transporters in sink tissues (Morita et al., 2009; Shitan et al., 2014) and perhaps nicotine levels in leaf tissues may be reduced, while levels in roots may be increased or unaffected.

One *Nicotiana* species—*N. alata*—synthesizes nicotine in the roots but is unable to translocate it to the xylem for transport to the leaves (Pakdeechanuan et al., 2012). Genetic studies involving hybrids between *N. alata* and the closely related *N. langsdorffii*, which does accumulate nicotine in leaf tissue, indicate that more than one dominant locus is involved in blocking transport of nicotine from the root to the xylem (Pakdeechanuan et al., 2012). The expression of *NtMATE1* and *NtMATE2* is also observed in the root (Pakdeechanuan et al., 2012). The identification of those genes controlling the dominant loci blocking nicotine transport will further our understanding of the long-distance transport of plant alkaloids.

In *C. japonica*, berberine is transported from the lateral roots to the rhizome (Fujiwara et al., 1993). Three berberine transporters have been identified and belong to the ATP-binding cassette (ABC) family (CjABCB1/CjMDR1, CjABCB2, and CjABCB3), with *CjABCB1* and *CjABCB2* localized in the plasma membrane and expressed in the rhizome, possibly playing a role in the uptake of berberine in the rhizome (Shitan et al., 2003, 2013). In transgenic *C. japonica*, where *CjABCB1* was suppressed, berberine accumulation in the root decreased (Shitan et al., 2005).

In *C. roseus*, catharanthine—which is coupled with vindoline to produce vinblastine and vincristine—is transported from the leaf epidermis to the leaf surface, resulting in spatial separation of catharanthine and vindoline (Roepke et al., 2010). An ABC transporter, CrTPT2, which is specifically expressed in the leaf epidermis, functions as a catharanthine exporter (Yu and De Luca, 2013). Virus induced gene silencing (VIGS) of the *CrTPT2* in *C. roseus* resulted in reduced catharanthine levels on the leaf surface and caused an increased in catharanthine-vindoline dimers within leaves, demonstrating that altered transport of MIA intermediates may alter biosynthesis of MIAs (Yu and De Luca, 2013).

It is evident that altered expression of alkaloid transporters is able to alter the accumulation of alkaloids, whether that be through changes in transport processes and/or regulation of alkaloid biosynthesis itself. Candidate transporters for altering alkaloid accumulation processes would, however, need a high degree of specificity in recognizing the target alkaloids in order to not alter other transport processes in the plant, which may have undesirable consequences. In the case of QA transport in lupin, the transporters involved would include plasma membrane located exporters in cells of aerial tissue, membrane-localized transporters for entry onto the phloem, plasma membrane importers in cells of reproductive tissue, and vacuolar membrane importers in cells of both aerial and reproductive tissue, as alkaloids are often sequestered within vacuoles to avoid toxic effects within tissues (Yazaki et al., 2008). The transporters of most interest for lupin breeding would be those involved in the import of QAs into the grain from the phloem, as QA levels in aerial tissue and phloem sap would ideally be high to deter feeding of chewing and sap-sucking insects.

## Genes Controlling QA Content in Lupins

### Lupin ‘Low Alkaloid’ Domestication Genes

In addition to QA biosynthetic genes, major loci controlling QA content are known. All modern lupin cultivars display a significantly lower QA phenotype compared with wild varieties due to ‘low-alkaloid’ domestication genes, specific for each lupin species with most arising from natural mutation. Low-alkaloid mutants of NLL, *L. luteus, L. albus*, and *L. mutabilis* were first identified in Germany in the late 1920s to early 1930s from wild germplasm (von Sengbusch, 1942) and give insights into the regulation of QA biosynthesis.

Natural low alkaloid mutations in NLL (*iucundus, esculentus*, and *depressus*) and *L. luteus (amoenus, dulcis*, and *liber*) are recessive, segregating independently of one another and follow a simple Mendelian inheritance pattern of clear 1:3 segregation (von Sengbusch, 1942; Gustafsson and Gadd, 1965). A fourth NLL locus *tantalus* was later identified by x-ray induced mutation (Zachow, 1967). The locus *iucundus* appears to have been exclusively used for NLL breeding and *dulcis* for *L. luteus* breeding (Lamberts, 1955; Gustafsson and Gadd, 1965; Gladstones, 1970). Of various, presumed natural, recessive low-alkaloid mutations in *L. albus*, identified by several authors, *pauper, mitis, reductus, exiguus*, and *nutricius* are located at different loci (Harrison and Williams, 1982; Kurlovich, 2002). The *pauper* locus is the most effective mutation in reducing QA levels and is now almost exclusively used in breeding programs, though *nutricius* and *exiguus* have been used in certain cultivars (Gladstones, 1970; Harrison and Williams, 1982). Low alkaloid material of *L. mutabilis* identified in the 1930s was lost (von Sengbusch, 1942), and it was not until other natural low alkaloid mutants were reselected over several generations, that the first sweet variety with grain content less than 0.05% was developed in the early 1980s (von Baer and Gross, 1977, 1983; Clements et al., 2008). The cultivar Inti was then produced which has a QA content less than 0.02% (Gross et al., 1988). Crosses between Inti and bitter *L. mutabilis* revealed inheritance of the low alkaloid trait is recessive, though F2 segregation is slightly higher than 1:4, indicating that the low alkaloid phenotype in Inti is controlled by a major, as well as additional minor alleles (Clements et al., 2008). Of the low-alkaloid mutations identified in lupin, none eliminates QAs completely (Gustafsson and Gadd, 1965; Gladstones, 1970; Harrison and Williams, 1982).

For low-alkaloid loci *dulcis* and presumably *pauper*, the limiting step of the QA pathway may be the reaction from cadaverine to the major structural QAs, as lysine and cadaverine levels do not differ between sweet and bitter plants in *L. luteus* and *L. albus*, nor do enzymatic activities for QA acyltransferases (Saito et al., 1993). Sweet NLL harboring the *iucundus* locus appears to have lower levels of lysine than bitter wild NLL, suggesting a different function for this gene (Bunsupa et al., 2012a). While these species of lupin cannot be crossed, the identification of low alkaloid genes will assist in further elucidating the QA pathway and will allow homologous genes between species to be identified and targeted in breeding programs. Markers linked to low alkaloid loci *iucundus* and *pauper* have been developed to assist tracking these recessive loci (Lin et al., 2009; Li et al., 2011). Recently, dense mapping resources, an updated genetic map for NLL cv. Tanjil and genome annotation have further narrowed the candidate gene region of *iucundus* on NLL-07 (Hane et al., 2016).

### Regulators of Alkaloid Biosynthesis

Jasmonic acid (JA) is a plant hormone regulating defense responses against environmental stresses and attack by pathogens and insects (Farmer et al., 2003) and is a well-known activator of alkaloid biosynthesis in *Nicotiana, C. roseus* and *C. japonica*; the expression of biosynthetic genes and alkaloid levels in nicotine, MIA and BIA biosynthesis, as well as previously mentioned *Nicotiana* and *C. roseus* alkaloid transporters, respond positively to JA treatment (Aerts et al., 1994; Pauli and Kutchan, 1998; Shoji et al., 2008, 2009; Morita et al., 2009; Yu and De Luca, 2013; Shitan et al., 2014; Gurkok et al., 2015; Kato et al., 2015). Many transcription factors regulating alkaloid biosynthesis, including basic Helix-Loop-Helix (bHLH), APELATA 2/Ethylene-Responsive Factor (AP2/ERF) and WRKY transcription factors identified in *Nicotiana, C. roseus*, and *C. japonica*, also show JA responsiveness (Menke et al., 1999; Chatel et al., 2003; Kato et al., 2007; Shoji et al., 2010; Todd et al., 2010; Suttipanta et al., 2011; Yamada et al., 2011; Van Moerkercke et al., 2015). These bHLH and AP2/ERF transcription factors regulate alkaloid biosynthesis by recognizing GCC-motif and G-box elements in the promoters of alkaloid biosynthetic genes in *Nicotiana* and *C. roseus* (Chatel et al., 2003; Shoji et al., 2010; De Boer et al., 2011), while a WRKY transcription factor binds to W-box elements in a *C. roseus* alkaloid biosynthetic gene promoter (Suttipanta et al., 2011). A *C. roseus* bHLH transcription factor can also bind to a G-box-like element in an AP2/ERF promoter which in turn promotes MIA biosynthesis (Zhang et al., 2011). As QA levels in lupin vegetative material is known to increase after wounding (Wink, 1983; Chludil et al., 2013), QA biosynthesis may be regulated by the JA pathway in similar ways to other alkaloids and it may be possible to identify similar candidate transcription factors regulating QA biosynthesis. More recently, microRNAs (miRNAs)—endogenous, small, non-coding RNAs which regulate gene expression by causing target mRNA degradation or translational repression (Naqvi et al., 2012)—have been identified which may regulate nicotine and BIA biosynthetic genes (Boke et al., 2015; Li et al., 2015). Nicotine biosynthesis can also be controlled by non-coding target mimicry (eTM)-mediated inhibition of its corresponding nicotine biosynthetic gene-targeting miRNA (Li et al., 2015). It will therefore be interesting to analyze the role of miRNAs in regulating QA biosynthesis in lupin. Putative miRNAs have been identified from *L. albus* phloem exudate (Rodriguez-Medina et al., 2011), and as additional lupin miRNA data sets become available, miRNAs regulating QA biosynthesis may be identified in order to better understand how the pathway is regulated.

## Environmental Factors Affecting QA Production

There is a significant environmental impact on grain QA content in lupin, due to either regulation of QA biosynthesis or transport from source tissues to the seed, though this impact appears to be highly unpredictable, with QA levels poorly explained by environmental properties such as location and seasonal climate (Cowling and Tarr, 2004). Grain QA levels can often exceed industry limits, usually by a couple of fold, though concentrations up to 2120 mg/kg have been found in sweet NLL, exceeding the limit by more than 10 times (Cowling and Tarr, 2004; Reinhard et al., 2006). There is, therefore, a great need to better understand how QA biosynthesis and transport is affected by environmental factors.

Light regulates QA biosynthesis by affecting the conditions within the chloroplast, with L/ODC activated by reduced thioredoxin and a light-mediated shift in pH of the chloroplast stroma from pH 7 to 8 during the day (Wink and Hartmann, 1981). As such, QA biosynthesis displays a diurnal rhythm whereby leaf QA concentrations are higher during the day and lower during the night (Wink and Witte, 1984). As light conditions cannot be controlled in the field, this factor is likely of less concern to breeders and farmers. Drought conditions are thought to increase QA content in lupin and drought stress can increase alkaloid levels in *Nicotiana, P. somniferum*, and *C. roseus* (Waller and Nowacki, 1978; Szabó et al., 2003; Jaleel et al., 2007). The effect of drought on grain QA content in lupin is not clear, with the plant growth stage at which drought is imposed seeming to play a role in whether QA content increases or decreases, albeit marginally (Christiansen et al., 1997), however, the amount of rainfall is not strongly associated with seed QA content (Cowling and Tarr, 2004). Ambient temperature seems to have an important effect on QA content, with a small increase in mean temperature (3°C) having a marked increase in grain QA content in European NLL varieties (Jansen et al., 2009, 2015). Soil characteristics, such as soil pH and the type and amount of fertilizer used, also affect grain QA levels. Higher soil pH (6.7 and 7.1) results in lower QAs than lower soil pH (5.3 and 5.8; Jansen et al., 2012). Potassium deficiency increases QAs, while phosphorus deficiency reduces them, with a significant interaction between potassium and phosphorous on QA content (Gremigni et al., 2001, 2003). The growing system also has a small effect on grain QA content, with organic conditions resulting in lower QA content than conventional conditions (Jansen et al., 2015). The amplitude of the response of grain QA content to environmental factors is also dependent on genotype, with some NLL cultivars more variable in QA content than others (Gremigni et al., 2000; Cowling and Tarr, 2004; Jansen et al., 2015).

While a few studies have investigated the role of abiotic stresses on QA biosynthesis, there are currently no reports of the impact of biotic stresses on grain QA content. As QAs play a role in the protection of the plant from predators, it is thought that QA accumulation may increase as part of a defense response when the plant comes under attack. Mechanical wounding of lupin leaves and plants, which may mimic herbivore action, has increased QA accumulation in vegetative material (Wink, 1983; Chludil et al., 2013). Leaf damage also leads to an increase in nicotine biosynthesis in *Nicotiana* (Baldwin, 1989; Cane et al., 2005). In a field situation, however, the large-scale wounding of lupin crops is not likely. Of greater concern to lupin growers are insect pests such as aphids, which can cause significant yield losses (Berlandier and Sweetingham, 2003). While it is known that QAs are a feeding deterrent to aphids (Berlandier, 1996; Ridsdill-Smith et al., 2004; Adhikari et al., 2012; Philippi et al., 2015), how QA production, and QA content in lupin grain respond to aphid attack has not yet been investigated. Additionally, the attack of lupin plants by fungal pathogens may impact QA production as alkaloid biosynthetic genes in *C. roseus* and *P. somniferum* are induced by treatment with fungal elicitors (Pasquali et al., 1992; Facchini et al., 1996a). It is likely that many different factors impact on QA biosynthesis in a field situation, and those most important need to be identified in order to be able to grow a valuable lupin crop.

## QA Quantification Methods

High or low grain QA phenotypes in lupin were first identified by a method similar to the still-employed Dragendorff test; the Dragendorff reagent reacts with high QA phenotypes (>0.5%), described as bitter, and low alkaloid phenotypes with no reaction are described as sweet (Harrison and Williams, 1982; Harborne, 1984). More accurate and nowadays the more common method of QA quantification is performed with gas-chromatography (sometimes gas-liquid chromatography) combined with a detector, usually a mass spectrometer. Most studies report on QA content in lupin grain and food products, though some studies also report on leaves and less commonly flowers, stems, roots and phloem sap (Gremigni et al., 2003; Cowling and Tarr, 2004; Reinhard et al., 2006; Lee et al., 2007; Resta et al., 2008; Hernández et al., 2011; Adhikari et al., 2012; Kamel et al., 2015). QA extraction is performed by leaching compounds from samples using an aqueous acidic solution, and then adjusting the solution to a basic pH for QA extraction with an organic solvent, usually dichloromethane (Wink et al., 1995; Ganzera et al., 2010). Wink et al. (1995) provide the most comprehensive spectral dataset for QAs, reporting Kovat’s indices and mass spectral data for 100 alkaloids found in different species of lupin. Many subsequent studies make use of this mass spectral data to identify QAs (Erdemoglu et al., 2007; Resta et al., 2008) as obtaining pure chemical standards of QAs is difficult as they are expensive and not readily available commercially. As a consequence, quantification has been based on relative concentrations of QAs (Erdemoglu et al., 2007; Chludil et al., 2009) or standard curves of major alkaloids (e.g., lupanine, gramine, or sparteine) or internal standards (e.g., caffeine, matrine) which are then applied to estimate concentrations of various other QAs (Muzquiz et al., 1994; Resta et al., 2008). Isolation of reference QAs from lupin tissue is also possible (Brooke et al., 1996; Wang et al., 2000; Reinhard et al., 2006) and has been used to quantify major QAs in NLL grain (Priddis, 1983; Harris and Wilson, 1988). Limit of detection (LOD) and limit of quantification (LOQ) values for the identification and quantification of QAs are not often reported, but for those that are, LODs range from 1 to 30.5 μg/mL and LOQs range from 3 to 87 μg/mL (**Table 2**) (Boschin et al., 2008; Resta et al., 2008; Ganzera et al., 2010). Despite many reports of quantification of QAs, many studies rely on relative QA quantifications or quantification for certain major QAs in mainly seed material of cultivated lupin species. In addition to the few major QAs, the presence of many minor QAs has been established in lupin leaf and grain (Wink et al., 1995) and levels of these may need further evaluation. Particularly in the case of cultivated NLL, which has a narrow genetic base and for which wild germplasm is often used as a source of genetic variation in pre-breeding material (Cowling et al., 2009; Berger et al., 2012, 2013), additional levels of minor QAs which are not being monitored may be inadvertently introduced by such breeding practices. There is, therefore, a need for an improved and more thorough methodology for the detection and quantification of QAs in lupin grain as well as other tissue types, for monitoring grain QA levels for food safety and to further facilitate the understanding of QA biosynthesis and accumulation.

**Table 2:** Limit of detection (LOD) and limit of quantification (LOQ) values for measurement of quinolizidine alkaloids by gas-chromatography mass-spectrometry (GC-MS) or capillary-electrophoresis mass-spectrometry (CE-MS) in lupin grain and lupin-based foods.

Compound	LOD (μg/mL)	LOQ (μg/mL)	Reference
Lupanine	2	3	Boschin et al., 2008; Resta et al., 2008
	7.9	18.2	Ganzera et al., 2010
Sparteine	1	3	Boschin et al., 2008; Resta et al., 2008
	30.5	87	Ganzera et al., 2010
13α-hydroxylupanine	6.5	12.5	Ganzera et al., 2010
Angustifoline	16.1	61.4	Ganzera et al., 2010

## Future Prospects

There is now a realistic opportunity for further elucidating the QA biosynthetic pathway in lupin grain crops and tackling the problem of QA accumulation for an emerging human health food. Recently developed genetic and genomic resources for NLL will greatly facilitate the identification of genes involved in this pathway, including the generation of a comprehensive NLL genome sequence (Hane et al., 2016), transcriptomic data sets for various NLL tissue types as well as three other cultivated lupin species (Parra-González et al., 2012; O’Rourke et al., 2013; Secco et al., 2014; Wang et al., 2014; Foley et al., 2015; Kamphuis et al., 2015), and dense genetic maps for NLL and *L. albus* (Croxford et al., 2008; Nelson et al., 2010; Yang et al., 2013; Kroc et al., 2014; Kamphuis et al., 2015). The remarkable progress in the elucidation of alkaloid biosynthetic pathways in model species in recent years, using a combination of genetic maps, genomic and transcriptomic resources, technical advances in enzymology, next generation sequencing, metabolite profiling and methodology for validating candidate genes with roles in alkaloid biosynthesis (i.e., RNAi or VIGS; Hagel and Facchini, 2013; Gurkok et al., 2015; Wang and Bennetzen, 2015; Kilgore and Kutchan, 2016; Pan et al., 2016), serves as a strong basis for understanding QA biosynthesis. Genetic and genomic resources can now be utilized in lupin to identify transcriptome-based candidate genes involved in QA biosynthesis and transport by comparative analysis between high and low QA varieties and plant tissue types or transcriptomic profiling analyzing QA-induced plants. The function of candidate genes may be studied by genetic transformation of lupin, the primary method being *Agrobacterium tumefaciens*-mediated transformation of wounded seedling shoot apical meristems to generate transgenic shoots (Pigeaire et al., 1997). While transformation efficiencies are low due to low survival and chimeric nature of T_0_ plants, this method has been successful in generating transgenic NLL (Molvig et al., 1997; Pigeaire et al., 1997; Wijayanto et al., 2009; Tabe et al., 2010; Atkins et al., 2011; Barker et al., 2016), *L. luteus* (Li et al., 2000; Pniewski et al., 2006), and *L. mutabilis* (Babaoglu et al., 2000; Polowick et al., 2014) to confer various traits, with recent modifications improving this transformation method for NLL (Nguyen et al., 2016a,b). For *L. albus*, however, *A. tumefaciens*-mediated transformation has been unsuccessful and as such hairy root transformation using *A. rhizogenes* (Uhde-Stone et al., 2005; Sbabou et al., 2010; Cheng et al., 2011) and VIGS using the *Peanut stunt virus* vector (Yamagishi et al., 2015) have been used to study gene function in this species. Metabolite profiling is an additional resource that could be enhanced in lupins to provide a valuable understanding of how the QA pathway interacts with other metabolic pathways in the plant, especially under abiotic and biotic stresses. This would be useful in understanding the effect of environmental and genotypic interactions on QA biosynthesis. The metabolite profiling of genetically diverse wild NLL accessions that will be used to introgress novel traits into pre-breeding lines would also be of interest as the level of genetic variation in QA content and composition in wild NLL is unclear and accessions which may introduce QAs which are currently not monitored for, need to be identified.

A better understanding of the genes involved in QA production and transport will allow for the management of QA grain content in various ways. For NLL, the first approach would be to focus on introgressing recessive alleles other than the *iucundus* locus (e.g., *esculentus, depressus*, and *tantalus*) into new varieties with the hypothesis that stacking these could reduce the QA content further and well-below the 0.02% threshold for use as a product for human consumption. A second approach would be to use various reverse-genetics approaches to identify genes involved in the biosynthesis, regulation or transport of QAs in order to reduce QA biosynthesis or transport to the grain. As most reduced QA content in lupins are the result of spontaneous mutations, and low QA loci are simply inherited and thus major genes controlling the pathway, it may be possible to find complete knock out mutants in QA biosynthetic genes as none of the current low QA mutations remove the QA phenotype completely. The targeting of QA transporter genes may also allow the QA content in foliar tissue to remain at high levels, but reduce or nullify the transport of QAs to the seed thereby still providing strong protection of the foliar tissue to insect predation, yet producing grain suitable for human consumption. A third approach would be the use of CRISPR/Cas9 to edit genes involved in QA regulation, synthesis or transport, thereby reducing grain QA content. The use of this technology will depend on whether its products are classified as genetically modified in the regulatory systems of different countries. In Australia, where the majority of the world’s lupin grain is produced, current legislation would class this as genetically modified, and CRISPR/Cas9 is therefore a less desirable approach for the improvement of lupin crops.

In stark contrast to most other crop species, lupins are only very recently domesticated and modern varieties have a narrow genetic base. The excellent genetic and genomic resources available for lupin now offer significant opportunities to ensure grain QA levels remain below the industry limit to improve the quality of this high protein grain legume.

## Author Contributions

KF wrote the manuscript with input from RF, KHMS, KBS, and LK.

## Conflict of Interest Statement

The authors declare that the research was conducted in the absence of any commercial or financial relationships that could be construed as a potential conflict of interest.

## Abbreviations

BIA: benzylisoquinoline alkaloid
ECT/EFT-LCT/LFT: ρ-coumaroyl-CoA/feruloyl-CoA: (+)-epilupinine/(–)-lupinine *O*-coumaroyl/feruloyltransferase
HMT/HLT: tigloyl-CoA:(–)-13α-hydroxymultiflorine/(+)-13α-hydroxylupanine *O*-tigloyltransferase
L/ODC: lysine/ornithine decarboxylase
MIA: monoterpenoid indole alkaloid
NLL: narrow-leafed lupin
QA: quinolizidine alkaloid
